# Efficacy and Safety of Venous Closure Devices for Femoral Venous Access in Interventional Cardiology: A Systematic Review and Meta-Analysis

**DOI:** 10.3390/jpm16070340

**Published:** 2026-06-24

**Authors:** Andrea Giovanni Parato, Vincenzo Mirco La Fazia, Marco Marino, Marcello Marchetta, Laura Colarocchio, Giovanni Albano, Francesco Pocelli, Emanuele Chiarazzo, Alessandro Di Francesco, Lorenzo Gerardi, Weili Marco Xu, Valerio Marongiu, Giuseppe Stifano, Andrea Natale

**Affiliations:** 1 Department of Biomedicine and Prevention, Division of Cardiology, University of Tor Vergata, 00133 Rome, Italy; andrea.parato231198@gmail.com (A.G.P.); laura.colarocchio@gmail.com (L.C.); weilimarco.xu@gmail.com (W.M.X.);; 2 Texas Cardiac Arrhythmia Institute, St David’s Medical Center, Austin, TX 78705, USA; 3 Department of Experimental Medicine, University of Tor Vergata, 00133 Rome, Italy; 4 Interventional Electrophysiology, Scripps Clinic, San Diego, CA 92037, USA; 5 School of Medicine, Case Western Reserve University, Cleveland, OH 44106, USA

**Keywords:** venous closure device, femoral access, atrial fibrillation, interventional cardiology

## Abstract

**Background:** Venous closure devices (VCDs) are being increasingly used after femoral venous access to facilitate recovery, but their comparative efficacy and safety versus manual compression or figure-of-eight suture remain uncertain. Because femoral venous access management is influenced by patient-related and procedural factors, VCDs may contribute to a more personalized postprocedural recovery strategy. **Objective:** Evaluation of the impact of VCDs on procedural recovery and vascular complications in patients undergoing cardiac procedures via femoral venous access. **Methods:** We systematically searched PubMed, Embase, and CENTRAL through to April 2025 for randomized controlled trials (RCTs) comparing VCDs with manual compression and/or figure-of-eight suture. Primary efficacy outcomes were time to hemostasis (TTH), time to ambulation (TTA), time to discharge (TTD), and time to discharge eligibility (TTDe). Safety outcomes were major and minor vascular complications. Risk of bias was assessed with RoB 2, and certainty of evidence assessed with GRADE. Random-effects models were used to pool standardized mean differences (SMDs) or risk ratios (RRs) with 95% confidence intervals (CIs). **Results:** Seven RCTs (*n* = 948) were included. VCDs use significantly reduced TTH (SMD: −1.00; 95% CI: −1.57 to −0.42) and TTA (SMD: −1.50; 95% CI: −2.42 to −0.58). TTD showed a non-significant trend favoring VCDs (SMD: −0.99; 95% CI: −2.13 to 0.15), while TTDe was consistently shorter with VCDs across three trials. Major vascular complications were rare and similar between groups (RR: 0.41; 95% CI: 0.09–1.89). Minor vascular complications were significantly reduced with VCDs (RR: 0.42; 95% CI: 0.22–0.79). **Conclusions:** In patients requiring femoral venous access for interventional cardiology procedures, VCDs improve time to hemostasis and ambulation and reduce minor vascular complications without increasing major events. These findings support VCDs as an effective and safe strategy for venous closure.

## 1. Introduction

Over the last decade, advances in interventional cardiology and cardiac electrophysiology have led to a substantial increase in catheter-based procedures, including atrial fibrillation (AF) ablation, left atrial appendage occlusion (LAAO), and transcatheter valve repair [1,2]. Consequently, greater emphasis has been placed on optimizing postprocedural care, particularly through early mobilization, reduction of access-site complications, and implementation of same-day discharge strategies [3,4].

Hemostasis after femoral venous access has traditionally relied on manual compression (MC) with prolonged bedrest, a strategy associated with patient discomfort, delayed ambulation, longer hospitalization, and increased resource use [5]. The figure-of-eight subcutaneous suture was introduced as an alternative, improving hemostasis but patients remained susceptible to bleeding complications, especially under uninterrupted anticoagulation or with large-bore venous access [6,7].

To overcome these limitations, venous closure devices (VCDs) have been developed, including suture-mediated systems (Perclose™ ProGlide™ and ProStyle™), collagen plug-based devices (VASCADE MVP), and hydrogel-based sealant systems (MYNX CONTROL™) [8,9,10,11,12]. These devices are based on different mechanisms: suture-mediated systems provide mechanical approximation of the venous access site, collagen-based devices promote extravascular sealing, and hydrogel-based systems create a temporary sealant barrier. In clinical practice, their rationale is to achieve faster haemostasis, reduce prolonged bedrest, facilitate earlier ambulation, and support same-day discharge after procedures. The main characteristics and mechanisms of the venous closure device platforms evaluated in the included trials are summarized in Supplementary Table S1.

Femoral venous closure should not be considered a “one-size-fits-all” component of postprocedural care. The optimal closure strategy may vary according to bleeding risk, frailty, and anticoagulation status, as well as procedural characteristics, including sheath size, number of venous access sites, device platform, and planned discharge pathway. From this perspective, VCDs may represent a practical tool for personalized access-site management, aiming to match the closure strategy to the expected procedural risk and recovery needs of the individual patient.

Despite their increasing use, evidence supporting the efficacy and safety of VCDs remains inconsistent. Although randomized trials have reported reductions in time to hemostasis, ambulation, and access-site complications, most studies were underpowered to assess infrequent but clinically relevant safety outcomes such as major vascular complications, and heterogeneity across devices, procedures, and endpoint definitions has limited interpretability [9,10,11,12].

This systematic review and meta-analysis provides a quantitative synthesis of randomized evidence on venous closure devices, aiming to better define their role in access-site management for contemporary interventional cardiology practice.

## 2. Methods

### 2.1. Study Design and Reporting Guidelines

This systematic review and meta-analysis was conducted in accordance with the PRISMA 2020 guidelines (Supplementary Materials S1) and the methodological recommendations of the Cochrane Handbook for Systematic Reviews of Interventions. The protocol was prospectively registered in PROSPERO (ID: 1020336).

### 2.2. Eligibility Criteria

We included studies that met all the following criteria: (1) RCTs; (2) comparing venous closure devices (VCDs) with standard closure techniques (MC and/or figure-of-eight suture (Fo8)) in adult patients undergoing interventional cardiology procedures requiring femoral venous access; (3) procedures including catheter ablation (e.g., atrial fibrillation or ventricular tachycardia), left atrial appendage occlusion, transcatheter valve repair (TEER), patent foramen ovale (PFO) closure, or leadless pacemaker; (4) reporting at least one relevant outcome as described below; and (5) providing follow-up through hospital discharge or up to 30 days. We excluded: (1) non-randomized or observational studies; (2) studies conducted in pediatric populations; (3) studies evaluating arterial access or closure, or those combining arterial and venous access without separate reporting of venous outcomes; and (4) studies with overlapping populations unless additional unique data were available.

### 2.3. Information Sources and Search Strategy

We systematically searched PubMed, Embase, and the Cochrane Central Register of Controlled Trials (CENTRAL) from database inception through to 30 April 2025. The search strategy combined controlled vocabulary (e.g., MeSH terms) and free-text keywords related to venous closure devices and interventional cardiology procedures. Free-text keywords included terms related to venous closure technologies and device names, such as “venous closure device”, “venous closure system”, “venous access closure”, “closure device”, “ProGlide”, “Perclose”, “ProStyle”, “Vascade”, and “Mynx”, combined with terms related to cardiac procedures, including “catheter ablation”, “atrial fibrillation”, “electrophysiology study”, “pulmonary vein isolation”, “leadless pacemaker”, and “left atrial appendage occlusion”. The full search strategy is reported in Supplementary Table S2.

### 2.4. Study Selection

**Figure 1:**
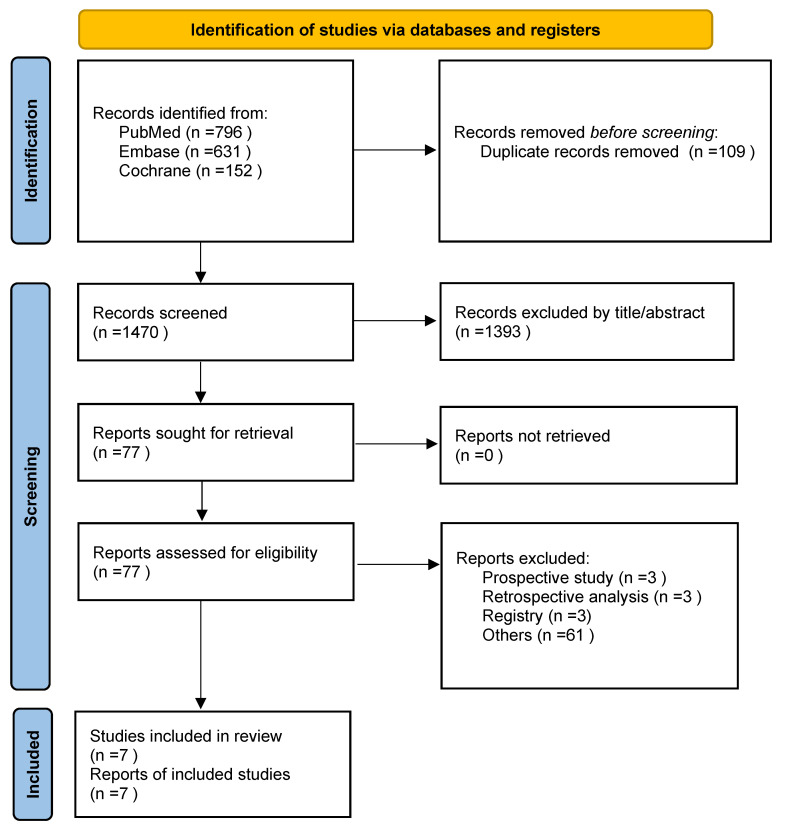
PRISMA 2020 flow diagram for updated systematic reviews which included searches of databases and registers only.

Two reviewers (G.A. and A.G.P.) independently screened titles, abstracts, and full texts in a two-step process. Disagreements were resolved through discussion or consultation with a third reviewer (M.M.). Reasons for exclusion were documented at the full-text screening stage. The study selection process is illustrated in the PRISMA flow diagram (Figure 1).

### 2.5. Data Collection Process

Data were extracted independently by two reviewers (A.G.P. and G.A.) using a standardized data extraction form. Extracted data included study characteristics, patient population, procedural setting, VCD type, comparator, outcome definitions, and results. Discrepancies were resolved through consensus.

### 2.6. Outcomes and Definitions

Outcomes were defined and harmonized across studies and based on standardized classifications. Primary efficacy outcomes included: TTH, time from sheath or device removal to clinically confirmed complete hemostasis; TTA, time from sheath/device removal to unassisted walking of at least 20 feet without rebleeding; TTDe, time to meet institutional criteria for safe discharge, with emphasis on access-site stability; TTD, interval from procedure end to actual hospital discharge. Safety outcomes were categorized as major and minor vascular complications. Major vascular complications (MVCs) were defined as any access-site event requiring surgical/percutaneous intervention, transfusion, hospitalization, or prolonged observation. These included pseudoaneurysm requiring treatment, arteriovenous (AV) fistula requiring treatment, access-site infection requiring antibiotics or hospitalization, bleeding requiring transfusion, or BARC ≥ 3 bleeding. Minor vascular complications (mVCs) were considered as non-severe events not requiring intervention, including hematoma > 6 cm, pseudoaneurysm not treated, AV fistula not treated, localized infection managed conservatively, minor bleeding, or access-site pain requiring analgesia.

### 2.7. Additional Variables

We extracted data on mean age, sex, comorbidities (e.g., diabetes, chronic kidney disease), type of procedure, closure device characteristics, sheath size, anticoagulation strategy, and discharge protocol, when available.

### 2.8. Risk of Bias Assessment

Risk of bias was assessed using the Cochrane RoB 2 tool (version dated 22 August 2019), evaluating five domains: randomization, deviations from intended interventions, missing data, outcome measurement, and selection of reported results. Two reviewers (A.G.P. and G.A.) independently assessed each study, with discrepancies resolved by consensus. An overall risk of bias judgment was assigned to each study (low risk, some concerns, or high risk), as reported in Supplementary Table S3.

### 2.9. Effect Measures

For continuous outcomes, standardized mean differences (SMDs) with 95% confidence intervals (CIs) were calculated. For dichotomous outcomes, risk ratios (RRs) were used. All pooled estimates were calculated using a DerSimonian and Laird random-effects model. Statistical heterogeneity was assessed using the I^2^ statistic. When studies reported continuous outcomes as medians with interquartile ranges or ranges, means and standard deviations were estimated using validated conversion methods [13,14]. All analyses were conducted using Review Manager (RevMan), version 7.2.0 (The Cochrane Collaboration, 2024) and R version 4.5.0.

### 2.10. Ethical Statement

This study is a systematic review and meta-analysis based exclusively on data extracted from previously published studies. No individual patient-level data were collected or analyzed. Therefore, ethical approval and informed consent were not required.

## 3. Results

### 3.1. Study Selection

A total of 1579 records were identified through electronic database searches (PubMed = 796, Embase = 631, Cochrane CENTRAL = 152). After removal of 109 duplicates, 1470 records were screened based on titles and abstracts. Of these, 77 full-text articles were retrieved and assessed for eligibility. Seventy studies were excluded for the following reasons: wrong study design (e.g., single-arm series, observational cohorts, or registries; *n* = 9), duplicate or overlapping populations, use of arterial rather than venous closure devices, lack of appropriate comparator or incomplete outcome data (*n* = 61). Seven randomized controlled trials were ultimately included in the final qualitative and quantitative synthesis: Natale et al., 2020 [9], Tilz et al., 2024 [15], Ali et al., 2024 [16], Castro-Urda et al., 2023 [17], Lodhi et al., 2023 [18], Kiani et al., 2024 [19], and Summer et al., 2025 [20]. For the Summer et al., 2025 [20] study, both the conference abstract (2024) and the full-text article (2025) were retrieved; only the randomized controlled trial version published in 2025 was used for data extraction. Similarly, for the Kiani et al., 2024 [19] study, two separate reports and one abstract were identified, and the more recent publication containing the most updated dataset was selected for inclusion. The study selection process is illustrated in the PRISMA flow diagram (Figure 1).

### 3.2. Study Population

The seven included randomized controlled trials were published between 2020 and 2025 and enrolled a total of 948 patients, of whom 512 were destinated to VCDs and 436 to conventional hemostasis strategies (MC with or without Fo8). Sample sizes ranged from 40 to 270 patients. All trials included adult patients undergoing interventional cardiology procedures requiring femoral venous access and reported at least one efficacy or safety outcome relevant to this analysis.

Catheter ablation for atrial fibrillation represented the predominant procedural indication, accounting for the entire study population in five trials and the majority of patients in the remaining studies. Two trials also included other electrophysiological procedures (atrial flutter, atrial tachycardia, supraventricular tachycardia) and structural heart interventions, such as LAAO, transcatheter edge-to-edge mitral valve repair, and leadless pacemaker implantation. No study included arterial access procedures.

Substantial variability was observed in venous access characteristics. Ablation-focused trials generally involved multiple femoral venous punctures (two to four access sites) using small- to mid-bore sheaths (6–12 Fr inner diameter), whereas large-bore access studies evaluated single-site closure following implantation of devices requiring larger introducers, including MitraClip (22–24 Fr) and Micra leadless pacemakers (23–27 Fr). In all trials, each patient received a single venous closure strategy applied to all access sites, ensuring consistent outcome assessment.

The evaluated closure systems included suture-mediated devices (Perclose ProGlide™ and ProStyle™), collagen-based devices (VASCADE MVP), and sealant-based systems (MYNX CONTROL™). Comparator strategies varied across studies and reflected institutional practice, consisting of manual compression alone or manual compression combined with Fo8 suture.

Across trials, mean patient age ranged from 61 to 83 years, with male representation between 36% and 67%. Cardiovascular risk factors, including hypertension, diabetes mellitus, and coronary artery disease, were variably reported. Anticoagulation use was common, particularly in ablation studies, with most patients receiving uninterrupted oral anticoagulation. Follow-up duration ranged from in-hospital assessment to 30 days post-procedure. Detailed baseline clinical and procedural characteristics are summarized in Supplementary Tables S1 and S2.

This variability in procedural indication, sheath diameter, number of venous access sites, anticoagulation exposure, and discharge protocols underscores the need for individualized selection of the venous closure strategy rather than uniform application of a single closure approach across all interventional settings.

### 3.3. Risk of Bias Assessment

Risk of bias was independently assessed by two reviewers using the Cochrane Risk of Bias 2.0 (RoB 2) tool, evaluating five domains: randomization process, deviations from intended interventions, missing outcome data, outcome measurement, and selection of reported results. For efficacy outcomes, one trial (Natale et al., 2020 [9]) was judged to have a low overall risk of bias, while the remaining six studies were rated as having moderate risk, mainly due to concerns related to open-label design, deviations from intended interventions, and outcome measurement.

For safety outcomes, a similar risk-of-bias profile was observed. Natale et al., 2020 [9] maintained a low risk of bias across all domains, whereas the other trials were generally rated as low risk, with moderate concerns in the domain of deviations from intended interventions, consistent with their open-label nature. One study (Castro-Urda et al., 2022 [17]) also showed moderate risk of bias in outcome measurement for safety endpoints. Detailed assessments are reported in Supplementary Table S3.

### 3.4. Efficacy Outcomes

**Figure 2:**
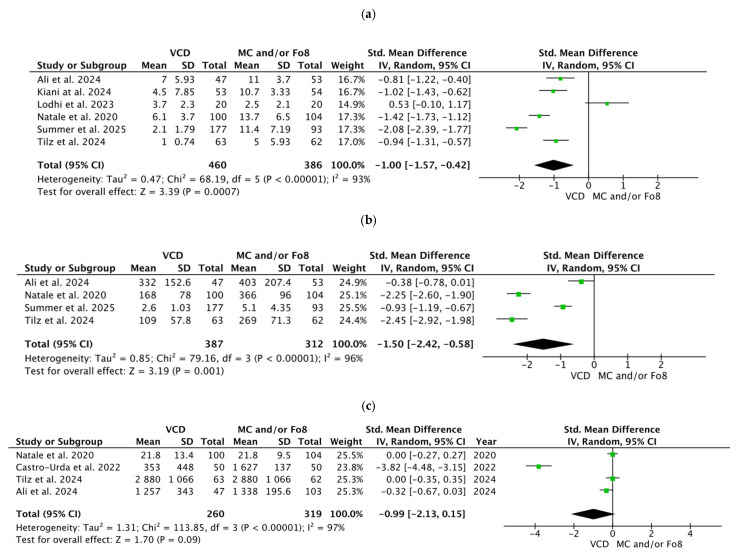
Forest plots showing TTH (**a**) [9,15,16,18,19,20], TTA (**b**) [9,15,16,20], and TTD (**c**) [9,15,16,17].

Time to hemostasis (TTH) was reported in five randomized controlled trials, including a total of 486 patients (VCD = 326; control = 160). The pooled analysis demonstrated a significant reduction in TTH in favor of the VCD group (SMD: −1.00; 95% confidence interval [CI]: −1.57 to −0.42; *p* = 0.0007), with substantial between-study heterogeneity (I^2^ = 93%) (Figure 2a).

TTA was evaluated in four randomized controlled trials (Tilz et al., 2024 [15], Summer et al., 2024 [20], Natale et al., 2020 [9], and Ali et al., 2024 [16]), including a total of 699 patients (387 in the VCD group and 312 in the control group). Meta-analysis demonstrated a significant reduction in TTA with VCD use (SMD: −1.50; 95% CI: −2.42 to −0.58; *p* = 0.001), indicating a large effect size. Substantial heterogeneity was observed across studies (I^2^ = 96%) (Figure 2b). TTD was reported in four studies (Natale et al., 2020 [9], Castro-Urda et al., 2023 [17], Ali et al., 2024 [16], and Tilz et al., 2024 [15]), including patients undergoing either electrophysiological or structural interventions. The pooled analysis showed a non-significant reduction in TTD in the VCD group compared with the control group (SMD: −0.99; 95% CI: −2.13 to 0.15; *p* = 0.09), with very high heterogeneity (I^2^ = 97%) (Figure 2c). Although not statistically significant, the observed trend suggests a possible benefit of VCDs in facilitating earlier discharge. TTDe was reported in three randomized controlled trials (Natale et al., 2020 [9], Tilz et al., 2024 [15], and Summer et al., 2024 [20]). Meta-analysis was not performed due to methodological limitations, including a zero standard deviation in the VCD arm of one study, which rendered the pooled estimate non-estimable. Results are therefore reported descriptively in the Section 3.6.

### 3.5. Safety Outcomes

**Figure 3:**
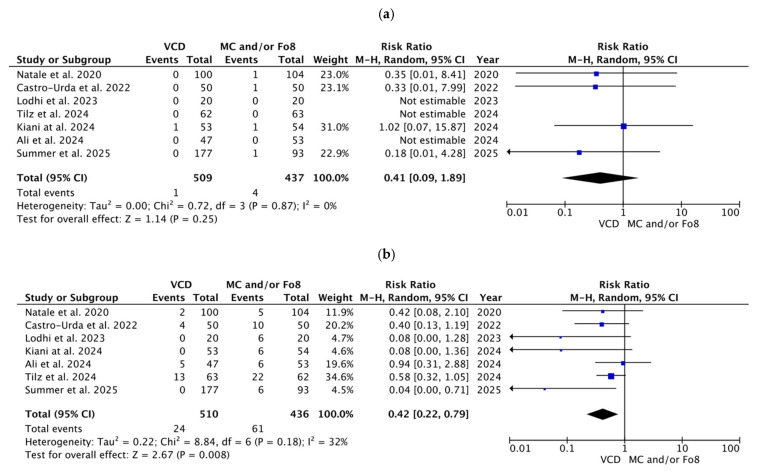
Forest plots showing (**a**) major vascular complications [9,15,16,17,18,19,20] and (**b**) minor vascular complications [9,15,16,17,18,19,20].

Major vascular complications were reported in seven randomized controlled trials and were defined using harmonized, prespecified criteria across studies. The total number of events was very low, with one major event in the VCD group (*n* = 509) and four events in the control group (*n* = 437). The pooled analysis showed a non-significant reduction in the risk of major vascular complications with VCD use (RR: 0.41; 95% CI: 0.09–1.89; *p* = 0.25), with no heterogeneity across studies (I^2^ = 0%) (Figure 3a). Although not statistically significant, the direction of effect consistently favored the VCD group, and the absence of heterogeneity supports the stability of the observed trend. All reported events were adjudicated based on meaningful clinical criteria, including the requirement for transfusion, surgical repair, or unplanned rehospitalization. These findings suggest that VCD use is not associated with an increased risk of serious vascular complications and may offer a favorable safety profile in appropriately selected patients.

Minor vascular complications were reported in all seven included trials and were classified according to standardized definitions encompassing access-site hematoma, bleeding, ecchymosis, or pain not requiring intervention. The pooled analysis showed a significant reduction in minor vascular complications among patients treated with VCDs (24 events in 510 patients) compared with standard closure methods (61 events in 436 patients) (RR: 0.42; 95% CI: 0.22–0.79; *p* = 0.008) (Figure 3b). Heterogeneity was low to moderate (I^2^ = 32%), with a consistent direction of effect across studies. These results support a clinically meaningful benefit of VCDs in reducing non-severe access-site-related events, which—although not life-threatening—may influence postprocedural recovery, patient comfort, and workflow efficiency in interventional cardiology settings. A detailed summary of reported major and minor vascular complications, together with the available information on their management, is provided in Supplementary Table S5.

### 3.6. Additional Analysis

Predefined sensitivity analyses were performed to assess the robustness of the primary efficacy outcomes and the potential influence of device type. The certainty of evidence for TTH and TTA was evaluated using the GRADE approach (Supplementary Table S4).

For TTH, the certainty of evidence was rated as moderate, reflecting concerns related to open-label designs and substantial statistical inconsistency. Sensitivity analyses confirmed the stability of the effect across device platforms. Exclusion of the sealant-based system (MYNX CONTROL™) or the collagen-based device (VASCADE MVP) did not materially change the direction or significance of the pooled effect. When analyses were restricted to suture-mediated devices (Perclose), the reduction in TTH remained statistically significant, although heterogeneity persisted (Supplementary Figure S1a–c).

Regarding TTA, the certainty of evidence was rated as low due to a serious risk of bias and very high heterogeneity. Sensitivity analyses excluding individual device types yielded comparable effect sizes, indicating a consistent advantage of venous closure devices in accelerating ambulation, despite persistent heterogeneity (Supplementary Figure S2a,b).

Primary analysis on TTD did not reach statistical significance and was characterized by very high heterogeneity. A sensitivity analysis excluding the study with identical discharge times between groups showed a numerically larger but still non-significant effect, underscoring the variability in discharge practices and endpoint definitions across trials (Supplementary Figure S3).

Lastly, for TTDe, quantitative pooling was not feasible due to methodological constraints, including zero variance in one study arm. Descriptive synthesis demonstrated a consistent direction of effect favoring venous closure devices across all contributing trials, suggesting improved access-site stability and earlier readiness for discharge.

These secondary and sensitivity analyses, together with funnel plots for efficacy and safety outcomes (Supplementary Figure S4), suggest that the clinical benefit of VCDs may be modulated by device type, procedural complexity, sheath size, and local recovery protocols. These findings are consistent with a personalized access-site management model in which closure strategy is selected according to procedural and patient-specific risk profiles.

## 4. Discussion

This systematic review and meta-analysis demonstrates that VCDs significantly improve key procedural recovery metrics, particularly TTH and TTA, compared with MC or Fo8 suture following femoral venous access in interventional cardiology procedures. These benefits were consistent in direction across all included randomized controlled trials and were supported by prespecified sensitivity analyses and GRADE-based assessments. Our findings extend those of the meta-analysis by Pang et al. [21], which focused primarily on arterial access, by providing venous-specific evidence relevant to contemporary electrophysiology and structural heart interventions.

From a clinical perspective, these findings are particularly relevant in settings where femoral venous access is frequently performed under uninterrupted anticoagulation and may involve multiple puncture sites or large-bore sheaths. In this context, even minor access-site events may delay mobilization, increase patient discomfort, require additional surveillance, or interfere with same-day discharge protocols. Therefore, the observed reductions in TTH, TTA, and minor vascular complications should be interpreted not only as procedural metrics, but also as clinically meaningful indicators of improved postprocedural recovery. Moreover, from a personalized medicine perspective, these findings suggest that femoral venous closure should be integrated into a patient- and procedure-tailored recovery pathway. Patients undergoing multiple venous punctures, large-bore venous access, uninterrupted anticoagulation, or procedures planned with same-day discharge protocols may derive particular benefit from closure strategies that shorten hemostasis and ambulation without increasing major vascular complications. Conversely, in lower-risk procedures or settings where early discharge is not planned, the incremental value of VCDs may depend more strongly on local workflow, device cost, and operator experience. Thus, the clinical role of VCDs should be interpreted within an individualized access-site management framework rather than as a uniform strategy for all patients.

The reduction in TTH associated with VCD use was both statistically and clinically meaningful. Subgroup analyses confirmed that this benefit was preserved across different device platforms, including suture-mediated, collagen-based, and sealant-based systems. Although substantial heterogeneity was observed, this likely reflects procedural and measurement differences rather than true inconsistency in treatment effect. The certainty of evidence for this endpoint was rated as moderate, supporting the robustness of the observed association.

The clinical plausibility of this benefit is supported by the mechanisms of the evaluated device platforms. Suture-mediated systems such as Perclose ProGlide™ and ProStyle™ provide mechanical approximation of the venous puncture site, whereas collagen-based devices such as VASCADE MVP and sealant-based systems such as MYNX CONTROL™ promote extravascular sealing without permanent intravascular material. These platforms also differ in their recommended sheath-size ranges: Perclose ProGlide™ and ProStyle™ are indicated for common femoral venous closure using 5F–24F sheaths, whereas VASCADE MVP and MYNX CONTROL Venous VCD are intended for 6F–12F inner-diameter venous sheaths. These characteristics may be particularly relevant as contemporary electrophysiology procedures are increasingly performed in complex populations and with expanding procedural strategies, including advanced approaches such as persistent AF ablation and concomitant left atrial appendage closure [22,23,24]. Although direct device-to-device comparisons remain limited, the consistency of benefit across sensitivity analyses suggests that improved venous hemostasis may represent a shared effect across platforms, while the magnitude of benefit may vary according to device type, sheath size, anticoagulation strategy, and local ambulation protocols. This reinforces the concept that device selection should be guided by procedural anatomy and clinical context, with suture-mediated, collagen-based, and sealant-based systems potentially addressing different access-site scenarios within a precision-oriented procedural workflow. Emerging data on VASCADE MVP-XL further suggest that dedicated collagen-based closure platforms may extend the applicability of extravascular venous closure to larger-bore electrophysiology procedures, although device-specific evidence remains limited [25]. Other emerging technologies, such as the Venock venous closure system, are also being developed for large-bore femoral venous access, but their role remains investigational pending peer-reviewed clinical validation [26].

In particular, the comparison between Perclose-based suture-mediated closure and figure-of-eight suture is clinically relevant because both strategies are commonly used after electrophysiology procedures. In the included randomized trials, suture-mediated closure was associated with faster recovery metrics than figure-of-eight suture, including shorter TTH and TTA in both STYLE-AF.

Angio-Seal™ VIP may reduce time to hemostasis, immobilization, and patient discomfort after antegrade common femoral artery puncture for popliteal and below-the-knee interventions. However, antegrade access in patients with peripheral arterial disease may be technically challenging because of puncture angles, vessel calcification, and small-caliber arteries. Therefore, Angio-Seal™ VIP should be used selectively after confirmation of suitable common femoral artery anatomy and anterior-wall puncture, preferably with ultrasound guidance [27].

Regarding sealant-based closure, MYNX CONTROL was associated with shorter TTH, TTA, and TTDe compared with manual compression, with 100% procedural/device success and no major or minor access-site complications in the VCD arm. These findings support the potential role of hydrogel-based sealant systems as non-suture venous closure strategies, although direct head-to-head comparisons with other VCD platforms remain unavailable [20].

Similarly, VCD use was associated with a significant reduction in TTA, although heterogeneity was very high. This variability appears largely driven by protocol-level differences between studies, particularly fixed post-procedural immobilization periods in control groups and non-uniform definitions of ambulation. Despite these limitations, the consistent direction of effect across trials supports the clinical relevance of earlier mobilization, especially in the context of same-day discharge pathways. From an economic perspective, shorter bedrest, earlier ambulation, and fewer minor access-site complications may improve patient throughput. However, available cost data remain mixed: the PROFA trial reported cost savings with Perclose ProGlide and early discharge, Kiani et al. found improved efficiency without increased overall procedural costs, whereas Lodhi et al. reported substantially higher direct closure costs with Perclose ProGlide compared with figure-of-eight suture. Therefore, the cost-effectiveness of VCDs likely depends on device pricing, discharge protocols, procedural volume, and patient selection. Although pooled analyses were performed using standardized mean differences because of heterogeneous reporting across trials, the absolute differences observed in individual studies were clinically meaningful. In AMBULATE, VASCADE MVP reduced mean TTH by 7.6 min per access site and TTA by approximately 3.3 h compared with manual compression, while in the ReliaSeal trial, MYNX CONTROL reduced TTH from 11.4 to 2.1 min and TTA from 5.1 to 2.6 h compared with manual compression.

This is particularly relevant given the growing adoption of same-day discharge protocols after atrial fibrillation ablation. Contemporary observational studies and meta-analyses have shown that same-day discharge can be feasible and safe in selected patients, with low rates of post-discharge complications, emergency department visits, and 30-day readmissions [28,29,30,31,32]. Similar principles have also been explored in selected patients undergoing uncomplicated transvenous lead extraction, further reinforcing the broader shift toward structured early-discharge pathways in electrophysiology [33]. In this context, reliable venous hemostasis is a key prerequisite for safe early mobilization, particularly in patients treated with uninterrupted anticoagulation and in high-volume centers. The VASCADE MVP postmarket registry further supports the feasibility of same-day discharge following catheter ablation when venous closure is incorporated into the procedural workflow [28]. Shorter bedrest, earlier ambulation, and fewer minor access-site events may also reduce nursing burden, improve patient throughput, and optimize recovery-area resources, although the present study was not designed as a formal cost-effectiveness analysis [34].

In contrast, TTD did not differ significantly between groups in the pooled analysis. This finding is likely explained by marked heterogeneity in discharge definitions and institutional workflows, which may obscure procedure-level effects. Although a numerical trend favoring VCDs was observed in several studies, these results suggest that discharge timing is highly context-dependent and influenced by non-clinical factors.

For TTDe, quantitative pooling was not feasible due to methodological constraints, including zero variance in one study arm. Nevertheless, all contributing trials showed a consistent direction of effect favoring VCD use, suggesting improved access-site stability and earlier readiness for discharge.

Evaluating safety endpoints, this meta-analysis provides reassuring evidence regarding the use of VCDs. Major vascular complications were infrequent, with no significant difference between VCDs and standard closure methods and no observed heterogeneity. Although the analysis was underpowered to detect small differences in rare events, the consistent direction of effect and absence of any safety signal support the use of VCDs for venous access-site management. Minor vascular complications were significantly reduced with VCD use, an effect that was consistent across studies and clinically relevant because access-site hematoma, oozing, ecchymosis, and local pain may delay ambulation, prolong observation, increase nursing surveillance, and reduce patient satisfaction despite not meeting criteria for major complications.

No meta-regression was performed due to the limited number of trials per outcome, as such analyses would have been underpowered and potentially misleading. Instead, subgroup and sensitivity analyses were used to explore the influence of device type, confirming the overall consistency of the findings.

The main strengths of this study include the inclusion of a relatively large, pooled population for this specific procedural setting, derived exclusively from randomized controlled trials, and the use of secondary and sensitivity analyses to explore the robustness of the findings and the potential influence of device type. In addition, the study provides a structured overview of the main venous closure platforms evaluated, including suture-mediated, collagen-based, and sealant-based systems, thereby offering operators a practical framework to understand the potential advantages and limitations of each device strategy.

Overall, these results support the role of venous closure devices as an effective strategy to enhance procedural recovery without compromising safety. Although heterogeneity remains for certain endpoints, particularly those influenced by institutional protocols, the consistent benefits observed for hemostasis and minor complication reduction highlight the value of VCDs in contemporary interventional cardiology practice.

## 5. Limitations

Several limitations should be acknowledged. The relatively limited sample size of the available randomized trials further reduced the statistical power to detect differences in rare safety outcomes, particularly major vascular complications. However, some events, such as arteriovenous fistula or retroperitoneal bleeding, are primarily related to vascular access rather than to the vascular closure device itself. Most included trials were open label, introducing potential performance and detection bias, particularly for subjective endpoints such as TTA. Although adjudication committees and prespecified definitions were used in several studies, blinding was generally not feasible and remains an inherent limitation.

Methodological heterogeneity, especially in the definition and measurement of efficacy endpoints, represents a major challenge. This was particularly evident for TTD, which was inconsistently defined across studies. While some trials measured TTD from sheath or device removal, others used exit from the electrophysiology laboratory or overall hospital length of stay, likely contributing to the very high heterogeneity observed (I^2^ = 97%) and limiting interpretability of this endpoint.

Additional heterogeneity arose from substantial variation in sheath size and number of venous access sites, ranging from multiple small-bore ablation sheaths to single large-bore introducers for leadless pacemaker implantation. Although procedural characteristics, including type of intervention, closure strategy, number of venous access sites, and sheath size, were extracted and summarized in Supplementary Table S2, the limited number of studies precluded dedicated subgroup analyses according to procedural type or sheath diameter. Antithrombotic management also varied across studies. Postprocedural antithrombotic adjustments were inconsistently reported and could not be systematically analyzed. Therefore, the potential influence of postprocedural medication changes on vascular complications or recovery outcomes cannot be excluded. Consequently, outcomes such as time to hemostasis and ambulation should be interpreted within the specific procedural and pharmacological context rather than as uniform effects across all interventions. Follow-up was limited to the early postprocedural period (generally ≤30 days), precluding assessment of delayed adverse events or long-term device durability. Although significant reductions were observed for time to hemostasis and ambulation, heterogeneity remained high, reflecting procedural differences in hemostasis assessment and protocol-driven immobilization strategies for ambulation.

Subgroup and sensitivity analyses were constrained by the small number of available studies for certain comparisons, particularly for time to discharge eligibility and device-specific analyses, reducing the precision of these estimates. Finally, the low incidence of major vascular complications limited statistical power for safety outcomes, an inherent limitation when evaluating rare events.

Despite these constraints, the consistent direction of effect across studies and the favorable safety profile observed support the clinical relevance of venous closure devices, particularly for improving procedural recovery and reducing minor access-site complications.

## 6. Conclusions

This systematic review and meta-analysis shows that VCDs significantly reduce TTH and TTA compared with MC or Fo8, without increasing major vascular complications. Importantly, VCDs were also associated with fewer minor vascular events, supporting their role as a safe and effective option for femoral venous closure in interventional cardiology. These findings support the use of VCDs as part of a personalized access-site management strategy in which closure technique is tailored to anticoagulation status, sheath size, number of venous access sites, procedural complexity, bleeding risk, and planned discharge pathway. Future studies should confirm these findings in broader procedural contexts and evaluate long-term and economic outcomes to better define which patients and procedures derive the greatest benefit from individualized VCD-based recovery protocols.

## Data Availability

No new data were created or analyzed in this study. All data supporting the findings of this systematic review and meta-analysis were extracted from previously published studies cited in the References section and are summarized in the main text and Supplementary Materials.

## Supplementary Materials

The following supporting information can be downloaded at: https://www.mdpi.com/article/10.3390/jpm16070340/s1. Figure S1: Forest plots showing a sensitivity analysis (excluding (a) Summer et al., 2025 and (b) Natale et al., 2020 and (c) both) for TTH; Figure S2: Forest plots showing a sensitivity analysis (excluding: (a) Summer et al., 2025 and (b) and Natale et al., 2020) for TTA; Figure S3: Forest plots showing a sensitivity analysis excluding: Natale et al., 2020 for TTD; Figure S4: Funnel plots for (A) TTH, (B) TTA, (C) TTD, (D) major vascular complications, and (E) minor vascular complications; Table S1: Characteristics and mechanisms of venous closure devices (VCDs); Table S2: Medical subject headings (MeSH) and non-MeSH keywords used to search for potential relevant publications; Table S3: Risk of bias summary for randomized studies (RoB2): (a) risk of bias for efficacy outcomes (b) risk of bias for safety outcomes; Table S4: GRADE summary of findings for efficacy endpoints (TTH, TTA); Table S5: Reported vascular complications and available management details across included randomized trials. Supplementary Materials S1: PRISMA 2020 Checklist.

## Abbreviations

AF	Atrial fibrillation
MC	Manual compression
Fo8	Figure of eight
RCT	Randomized controlled trial
TTA	Time to ambulation
TTD	Time to discharge
TTDe	Time to discharge eligibility
TTH	Time to hemostasis
VCD	Venous closure device
