# Supplementary material for: Efficacy and Safety of Venous Closure Devices for Femoral Venous Access in Interventional Cardiology: A Systematic Review and Meta-Analysis
Source: J Pers Med. 2026 Jun 24;16(7):340. doi: 10.3390/jpm16070340 (PMC13412252; doi:10.3390/jpm16070340)
Supplement: Supplementary file 1 [file jpm-16-00340-s001.zip › jpm-4252724-supplementary.pdf]

Supplementary materials

Supplementary Table S1. Characteristics and mechanisms of venous closure devices (VCDs).

Device	Type	Mechanism	Procedural use	Practical relevance
**Perclose ProGlide / ProStyle**	Suture-mediated closure	Mechanical approximation of the venous access site using a pre-deployed suture	AF ablation and large-bore venous access procedures	May facilitate rapid hemostasis after multiple or large-bore venous punctures
**VASCADE MVP**	Extravascular collagen-based closure system	Temporary intravascular disc assists positioning; extravascular collagen patch promotes sealing	Multi-access electrophysiology procedures	Designed to reduce time to hemostasis and ambulation without permanent intravascular material
**MYNX CONTROL**	Extravascular sealant-based closure system	Extravascular hydrogel/sealant barrier promotes access-site sealing	Electrophysiology procedures requiring femoral venous access	Non-suture strategy potentially useful for early ambulation and workflow optimization

Supplementary Table S2. Medical subject headings (MeSH) and non-MeSH keywords used to search for potential relevant publications

**Concept 1 (population)**	"vascular closure device"[Mesh] OR "venous closure device" OR "venous closure devices" OR "venous closure system" OR "venous access closure" OR "closure patch" OR "venous closure" OR "closure device" OR ("ProGlide" OR "Vascade" OR "MynxGrip" OR "AngioSeal" OR "Perclose" OR "Prostyle" OR "FamoSeal" OR "Manta" OR "StarClose" OR "VasoSeal" OR "ExoSeal" OR "InSeal" OR "PerQseal")
**Concept 2 (intervention)**	("catheter ablation" OR "atrial fibrillation" OR "arrhythmias, cardiac" OR "electrophysiologic techniques, cardiac" OR ablation OR "electrophysiology study" OR "electrophysiological study" OR "electrophysiological test" OR "electrophysiological testing" OR "mapping study" OR "electrophysiological mapping" OR "intracardiac mapping" OR "cardiac arrhythmia" OR "electrical stimulation" OR "intracardiac electrogram" OR "electrogram" OR arrhythmia OR tachycardia OR "conduction disorder" OR "radiofrequency ablation" OR "cryoablation" OR "pulmonary vein isolation" OR "substrate mapping" OR "electrophysiological procedures" OR pacemaker OR defibrillator OR "leadless pacemaker" OR "left atrial appendage occlusion" OR "laac")

Supplementary Table S3: Risk of bias summary for randomized studies (RoB2): (a)risk of bias for efficacy outcomes (b) risk of bias for safety outcomes

(a)

Study	Bias from randomization process	Bias due to deviations from intended interventions	Bias due to missing outcome data	Bias in measurement of the outcomes	Bias in selection of the reported result	Overall risk of bias judgement
Natale et al. 2020	Low	Low	Low	Low	Low	Low
Tilz et al. 2024	Low	Moderate	Low	Moderate	Low	Moderate
Ali et al. 2024	Low	Moderate	Low	Moderate	Low	Moderate
Castro-Urda et al. 2022	Low	Moderate	Low	Moderate	Low	Moderate
Lodhi et al. 2023	Low	Moderate	Low	Moderate	Low	Moderate
Kiani et al. 2024	Low	Moderate	Low	Moderate	Low	Moderate
Summer et al. 2024	Low	Moderate	Low	Moderate	Low	Moderate

(b)

Study	Bias from randomization process	Bias due to deviations from intended interventions	Bias due to missing outcome data	Bias in measurement of the outcomes	Bias in selection of the reported result	Overall risk of bias judgement
Natale et al. 2020	Low	Low	Low	Low	Low	Low
Tilz et al. 2024	Low	Moderate	Low	Low	Low	Low
Ali et al. 2024	Low	Moderate	Low	Low	Low	Low
Castro-Urda et al. 2022	Low	Moderate	Low	Moderate	Low	Moderate
Lodhi et al. 2023	Low	Moderate	Low	Low	Low	Low
Kiani et al. 2024	Low	Moderate	Low	Low	Low	Low
Summer et al. 2024	Low	Moderate	Low	Low	Low	Low

Supplementary Table S4. GRADE summary of findings for efficacy endpoints (TTH, TTA)

Certainty assessment							№ of patients		Effect		Certainty	Importance
№ of studies	Study design	Risk of bias	Inconsistency	Indirectness	Imprecision	Other considerations	VCD	MC and/or Fo8	Relative (95% CI)	Absolute (95% CI)		
**TTH**												
6	randomised trials	not serious	serious[a]	not serious	not serious	none	460	386	-	SMD –1.00 [–1.57 to –0.42]	⨁⨁⨁◯ **Moderate**	
**TTA**												
4	randomised trials	not serious	very serious[b]	not serious	not serious	none	387	312	-	SMD –1.50 [–2.42 to –0.58])	⨁⨁◯◯ **Low**	

CI: confidence interval; **SMD:** standardised mean difference

Explanations

a. Moderate to high heterogeneity (I^2 = 93%), with consistent direction of effect across all studies. Variability likely reflects procedural differences in the timing and definition of hemostasis assessment.
b. I^2 = 96%; high heterogeneity across studies with different ambulation definitions and fixed observation periods in control groups

Supplementary Figure S1. Forest plots showing a sensitivity analysis (excluding (a) Summer et al., 2025 and (b) Natale et al., 2020 and (c) both) for TTH

(a)

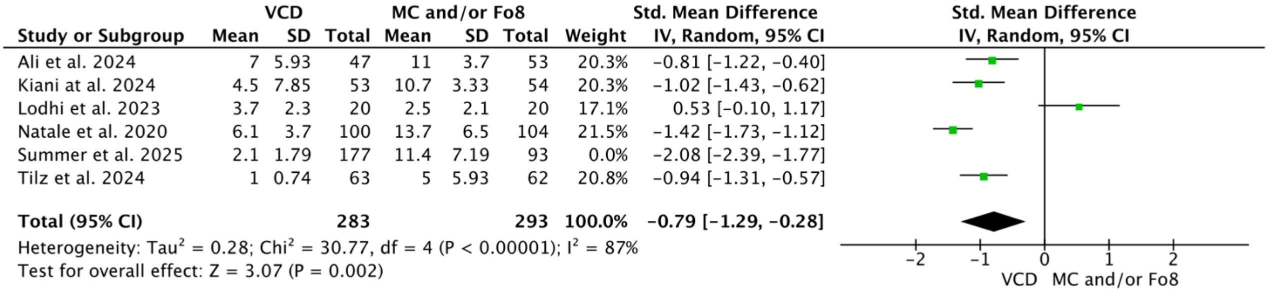

(b)

Study or Subgroup	VCD Mean	VCD SD	VCD Total	MC and/or Fo8 Mean	MC and/or Fo8 SD	MC and/or Fo8 Total	Weight	Std. Mean Difference IV, Random, 95% CI
Ali et al. 2024	7	5.93	47	11	3.7	53	20.2%	−0.81 [−1.22, −0.40]
Kiani at al. 2024	4.5	7.85	53	10.7	3.33	54	20.2%	−1.02 [−1.43, −0.62]
Lodhi et al. 2023	3.7	2.3	20	2.5	2.1	20	18.5%	0.53 [−0.10, 1.17]
Natale et al. 2020	6.1	3.7	100	13.7	6.5	104	0.0%	−1.42 [−1.73, −1.12]
Summer et al. 2025	2.1	1.79	177	11.4	7.19	93	20.7%	−2.08 [−2.39, −1.77]
Tilz et al. 2024	1	0.74	63	5	5.93	62	20.4%	−0.94 [−1.31, −0.57]
**Total (95% CI)**			**360**			**282**	**100.0%**	**−0.90 [−1.63, −0.16]**

Heterogeneity: $Tau^2 = 0.65$; $Chi^2 = 66.55$, df = 4 ($P < 0.00001$); $I^2 = 94\%$

Test for overall effect: $Z = 2.39$ ($P = 0.02$)

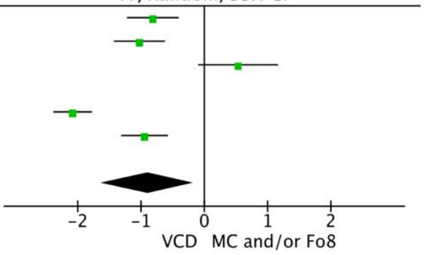

(c)

Study or Subgroup	VCD Mean	VCD SD	VCD Total	MC and/or Fo8 Mean	MC and/or Fo8 SD	MC and/or Fo8 Total	Weight	Std. Mean Difference IV, Random, 95% CI
Ali et al. 2024	7	5.93	47	11	3.7	53	25.9%	−0.81 [−1.22, −0.40]
Kiani at al. 2024	4.5	7.85	53	10.7	3.33	54	26.0%	−1.02 [−1.43, −0.62]
Lodhi et al. 2023	3.7	2.3	20	2.5	2.1	20	21.6%	0.53 [−0.10, 1.17]
Natale et al. 2020	6.1	3.7	100	13.7	6.5	104	0.0%	−1.42 [−1.73, −1.12]
Summer et al. 2025	2.1	1.79	177	11.4	7.19	93	0.0%	−2.08 [−2.39, −1.77]
Tilz et al. 2024	1	0.74	63	5	5.93	62	26.6%	−0.94 [−1.31, −0.57]
**Total (95% CI)**			**183**			**189**	**100.0%**	**−0.61 [−1.16, −0.06]**

Heterogeneity: $Tau^2 = 0.26$; $Chi^2 = 18.77$, df = 3 ($P = 0.0003$); $I^2 = 84\%$

Test for overall effect: $Z = 2.19$ ($P = 0.03$)

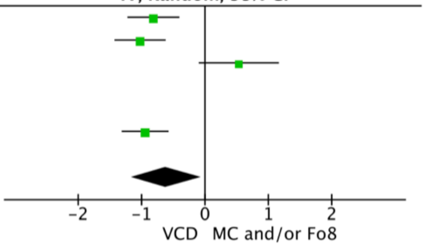

Supplementary Figure S2. Forest plots showing a sensitivity analysis (excluding: (a) Summer et al., 2025 and (b) and Natale et al., 2020) for TTA.

(a)

Study or Subgroup	VCD Mean	VCD SD	VCD Total	MC and/or Fo8 Mean	MC and/or Fo8 SD	MC and/or Fo8 Total	Weight	Std. Mean Difference IV, Random, 95% CI
Ali et al. 2024	332	152.6	47	403	207.4	53	33.4%	−0.38 [−0.78, 0.01]
Natale et al. 2020	168	78	100	366	96	104	33.6%	−2.25 [−2.60, −1.90]
Summer et al. 2025	2.6	1.03	177	5.1	4.35	93	0.0%	−0.93 [−1.19, −0.67]
Tilz et al. 2024	109	57.8	63	269	71.3	62	33.0%	−2.45 [−2.92, −1.98]
**Total (95% CI)**			**210**			**219**	**100.0%**	**−1.69 [−2.98, −0.40]**

Heterogeneity: $Tau^2 = 1.26$; $Chi^2 = 61.67$, df = 2 ($P < 0.00001$); $I^2 = 97\%$

Test for overall effect: $Z = 2.57$ ($P = 0.01$)

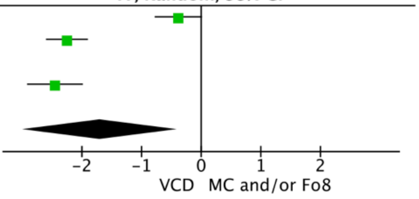

(b)

Study or Subgroup	VCD Mean	VCD SD	VCD Total	MC and/or Fo8 Mean	MC and/or Fo8 SD	MC and/or Fo8 Total	Weight	Std. Mean Difference IV, Random, 95% CI
Ali et al. 2024	7	5.93	47	11	3.7	53	20.2%	−0.81 [−1.22, −0.40]
Kiani at al. 2024	4.5	7.85	53	10.7	3.33	54	20.2%	−1.02 [−1.43, −0.62]
Lodhi et al. 2023	3.7	2.3	20	2.5	2.1	20	18.5%	0.53 [−0.10, 1.17]
Natale et al. 2020	6.1	3.7	100	13.7	6.5	104	0.0%	−1.42 [−1.73, −1.12]
Summer et al. 2025	2.1	1.79	177	11.4	7.19	93	20.7%	−2.08 [−2.39, −1.77]
Tilz et al. 2024	1	0.74	63	5	5.93	62	20.4%	−0.94 [−1.31, −0.57]
**Total (95% CI)**			**360**			**282**	**100.0%**	**−0.90 [−1.63, −0.16]**

Heterogeneity: $Tau^2 = 0.65$; $Chi^2 = 66.55$, df = 4 ($P < 0.00001$); $I^2 = 94\%$

Test for overall effect: $Z = 2.39$ ($P = 0.02$)

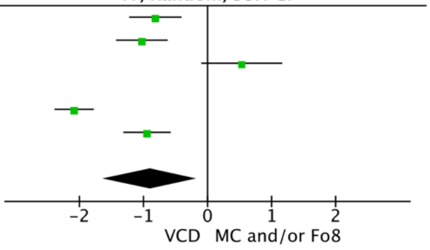

Supplementary Figure S3. Forest plots showing a sensitivity analysis excluding: Natale et al., 2020 for TTD.

	VCD			MC and/or Fo(				Std. Mean Difference	
Study or Subgroup	Mean	SD	Total	Mean	SD	Total	Weight	IV, Random, 95% CI	Year
Natale et al. 2020	21.8	13.4	100	21.8	9.5	104	0.0%	0.00 [−0.27, 0.27]	2020
Castro–Urda et al. 2022	353	448	50	1,627	137	50	32.6%	−3.82 [−4.48, −3.15]	2022
Tilz et al. 2024	2,880	1,066	63	2,880	1,066	62	33.7%	0.00 [−0.35, 0.35]	2024
Ali et al. 2024	1,257	343	47	1,338	195.6	103	33.7%	−0.32 [−0.67, 0.03]	2024
**Total (95% CI)**			**160**			**215**	**100.0%**	**−1.35 [−3.11, 0.41]**	

Heterogeneity: $Tau^2 = 2.35$; $Chi^2 = 103.06$, df = 2 ($P < 0.00001$); $I^2 = 98\%$
Test for overall effect: Z = 1.51 (P = 0.13)

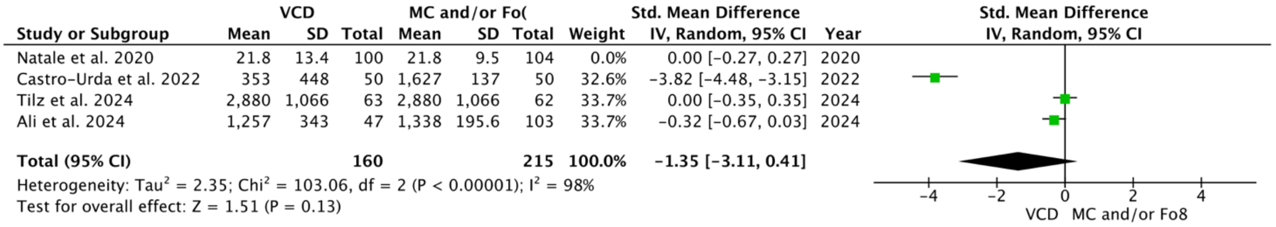

Supplementary Figure S4A- Funnel plot for Time to Hemostasis (TTH)

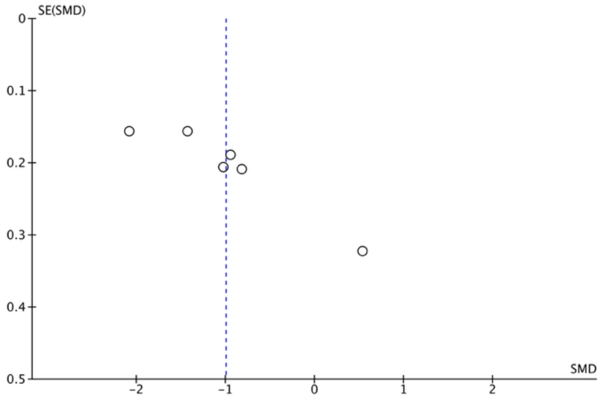

Supplementary Figure S4B- Funnel plot for Time to Hemostasis (TTA)

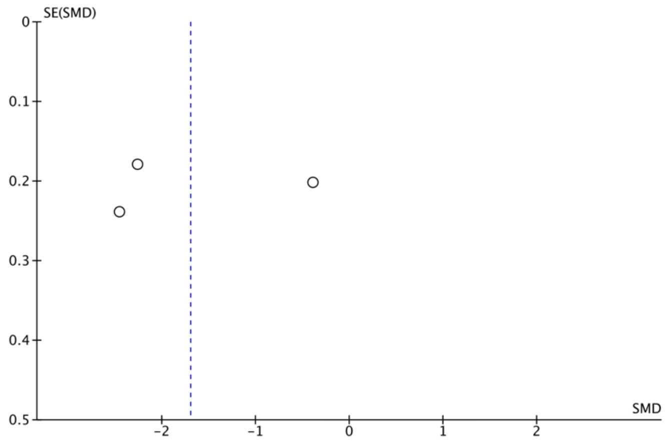

Supplementary Figure S4C- Funnel plot for Time to Hemostasis (TTD)

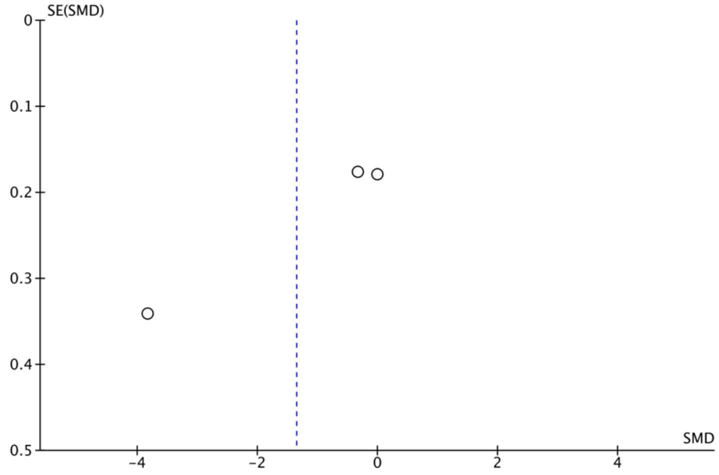

Supplementary Figure S4D- Major vascular complications

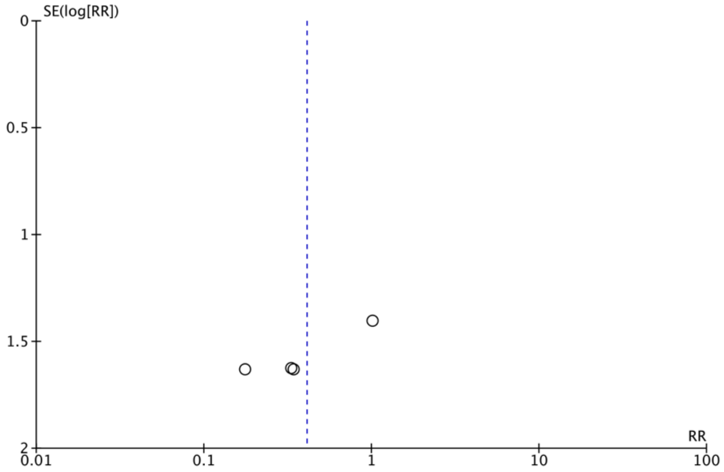

Supplementary Figure S4E- Minor vascular complications

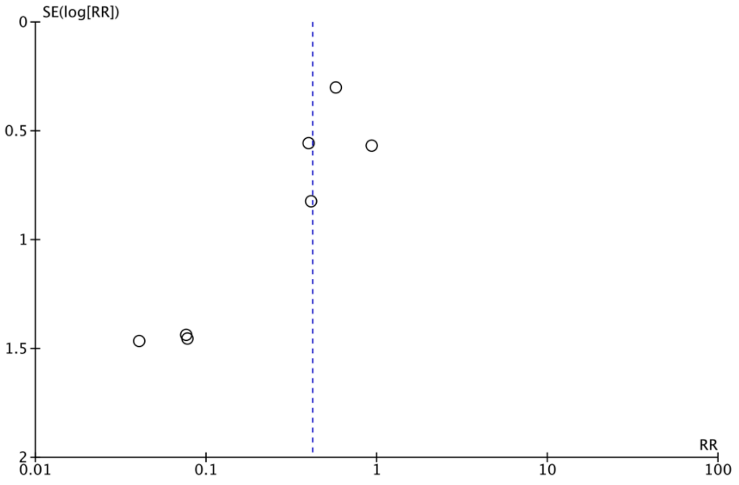

Supplementary Table S5. Reported vascular complications and available management details across included randomized trials

Study	Closure strategy	Major vascular complications	Management of major complications	Minor vascular complications	Management of minor complications	Notes
**Natale et al. 2020 (AMBULATE)**	VASCADE MVP vs manual compression	None in either group	N/A	VCD: 2 minor complications; MC: 5 minor complications	Not individually reported in the main text	Minor complications were adjudicated as access-site closure-related events. Ultrasound substudy showed no clinically significant safety issues.
**Tilz et al. 2024 (STYLE-AF)**	Perclose ProGlide/ProStyle vs figure-of-eight suture + manual compression	None in either group	N/A	Day of procedure: VCD 7/63 vs Fo8 15/62 patients with minor vascular complications. Events included groin hematoma >6 cm, groin hematoma <6 cm, and bleeding.	Mostly conservative observation; one VCD patient and one Fo8 patient had ambulatory medical evaluation for hematoma, with no intervention reported for the VCD patient	Groin hematoma <6 cm was more frequent in the Fo8 group. No major vascular access-related complications were reported at discharge or follow-up.
**Ali et al. 2024 (SAFE-VEIN)**	Perclose ProGlide vs figure-of-eight suture	None in either group	N/A	Minor bleeding: PPG 5 vs Fo8 4; hematoma: PPG 0 vs Fo8 2	Supplemental manual/mechanical compression was used when needed; FemoStop use was more frequent in the Fo8 group	No major bleeding, vascular thrombosis, dissection, pseudoaneurysm, arteriovenous fistula, vascular-related mortality, or other major vascular complications were reported.
**Castro-Urda et al. 2023 (PROFA)**	Perclose ProGlide and early discharge vs standard	Strategy A: 1 access-site bleeding event associated with hypotension	Strategy A: coronary angiography performed, no coronary stenosis,	Strategy A: access-site bleeding contributed to delayed	Strategy A: clinical evaluation and hospital observation; Strategy B:	No complications were considered directly related to Perclose ProGlide

	closure/overnight stay	and troponin rise; Strategy B: 1 arteriovenous fistula	type II myocardial infarction diagnosed; Strategy B: arteriovenous fistula treated with stent	discharge in one patient; Strategy B: access-site pain led to admission	admission for access-site pain	use by the study authors.
**Lodhi et al. 2023**	Perclose ProGlide vs figure-of-eight suture	Blood transfusion: Perclose 1 vs Fo8 1	Transfusion reported; further procedural management not specified	Composite access-site events: Perclose 4/20 vs Fo8 7/20. Events included ecchymosis >5 cm, hematoma, need for manual hold, ipsilateral venous ultrasound, access-site pain, and infection	Manual hold when required; ipsilateral venous ultrasound when required; other management not reported	None
**Kiani et al. 2024**	Suture-mediated closure vs manual compression	Major hematoma at discharge: SMC 2/53 vs MC 0/54; major hematoma at follow-up: SMC 0/51 vs MC 1/54	Management not individually reported; unplanned encounters for rebleeding or hematoma were recorded	Minor complications at discharge: SMC 4/51 vs MC 10/54; minor hematoma: SMC 1 vs MC 1; minor rebleeding/ooze: SMC 3 vs MC 10. Minor complications at follow-up: SMC 0/51 vs MC 2/53	Event-specific management not reported	No pseudoaneurysm, retroperitoneal bleeding, hemorrhage, AV fistula, venous thromboembolism, or access-site infection was reported.
**Summers et al. 2025 (ReliaSeal)**	MYNX CONTROL Venous VCD vs manual compression	VCD: none; MC: 1 access-site bleeding event requiring transfusion and surgical intervention	MC event required ICU monitoring; the patient was discharged in stable condition 3 days after the procedure	VCD: none; MC: 5% minor complication rate	Event-specific management not individually reported	Procedural and device success was 100% in the VCD group. No major or minor complications occurred in the VCD arm.

AV = arteriovenous; Fo8 = figure-of-eight suture; ICU = intensive care unit; MC = manual compression; PPG = Perclose ProGlide; SMC = suture-mediated closure; VCD = venous closure device.
